# 苯达莫司汀联合利妥昔单抗治疗复发滤泡性淋巴瘤的临床分析

**DOI:** 10.3760/cma.j.issn.0253-2727.2022.06.012

**Published:** 2022-06

**Authors:** 芳 赵, 莉莉 程, 重 郑, 慕晨 张, 澍 程, 彭鹏 许, 黎 王, 维莅 赵

**Affiliations:** 上海血液学研究所，医学基因组学国家重点实验室，国家转化医学中心（上海），上海交通大学医学院附属瑞金医院血液科，上海 200025 Shanghai Institute of Hematology, State Key Laboratory of Medical Genomics, National Research Center for Translational Medicine at Shanghai, Ruijin Hospital, Shanghai Jiao Tong University School of Medicine, Shanghai 200025, China

滤泡性淋巴瘤（FL）是较为常见的小B细胞来源的非霍奇金淋巴瘤（NHL）。免疫化疗方案R-CHOP（利妥昔单抗+环磷酰胺+多柔比星+长春新碱+泼尼松）/R-CVP（利妥昔单抗+环磷酰胺+长春新碱+泼尼松）的应用使初治FL患者获得了较好的疗效。国内外研究报道应用R-CHOP样免疫化疗方案能够使40％～76％的FL患者达到完全缓解（CR），总有效率超过90％[1]–[2]。但目前Ⅲ/Ⅳ期患者仍不能治愈，随着复发次数的增加，R-CHOP方案的疗效显著下降[3]。

在临床预后分层方面，国际多中心临床研究指出免疫化疗后2年内进展（POD24）的FL患者预后较差[4]。也有学者指出FL国际预后指数2（FLIPI2）高危（3～5分）的患者预后差[5]–[6]。另外，纳入7个基因（EZH2、ARID1A、MEF2B、EP300、FOXO1、CREBBP和CARD11）的m7-FLIPI的预后模型指出，在免疫化疗时代，ARID1A、MEF2B、EP300、FOXO1、CREBBP和CARD11基因突变患者的预后较未突变者差[7]。

复发和难治性FL在国际上尚无标准治疗方案，MacDonald等[8]探索了多种治疗复发难治性FL的方案，包括BR方案（苯达莫司汀联合利妥昔单抗），其总应答率为82％，中位无进展生存（PFS）时间为34.2个月，中位总生存（OS）时间长达109.7个月。日本一项Ⅱ期临床试验应用BR方案治疗37例复发或难治性FL（所有患者既往均接受过含利妥昔单抗的化疗，83.8％患者既往接受过R-CHOP方案治疗），总有效率为91.9％（95％*CI* 78.1％～98.3％），CR率为86.5％（95％ *CI* 71.2％～95.5％）。中位随访时间为39.5个月，3年PFS率和OS率分别为70.9％（95％*CI* 52.3％～83.3％）和88.9％（95％*CI* 73.1％～95.7％）[9]。因此，BR方案在国内外多个指南中被推荐作为复发FL的优选方案[10]。

BR方案治疗复发FL尚有一些需要解决的问题，如前序免疫化疗方案、POD24和肿瘤基因突变情况是否影响疗效等。本研究回顾性分析28例接受BR方案治疗的复发FL患者的临床资料，探讨影响患者生存的预后因素。

## 病例与方法

1. 病例：纳入2019年12月至2021年1月上海交通大学医学院附属瑞金医院血液科收治的28例复发或进展FL患者，所有患者在临床判断复发或进展时再次进行了肿瘤组织活检，明确诊断为FL（1～2级或3a级）。收集患者的临床资料，包括性别、年龄、FLIPI2评分，疾病分期、病理类型、有无B症状、外周血乳酸脱氢酶（LDH）、结外器官受累情况、骨髓受累情况、复发距末次治疗时间、血β_2_-微球蛋白（β_2_-MG）、BCL-2荧光原位杂交（FISH）结果。病理诊断均符合世界卫生组织（WHO）2016年淋巴组织肿瘤分类标准[11]。应用靶向测序方法对患者的肿瘤组织进行基因检测[12]。

2. 治疗方案与疗效：BR方案（利妥昔单抗375 mg/m^2^，第0天；苯达莫司汀90 mg/m^2^，第1～2天），每28 d为一个疗程。患者完成3、6个疗程BR方案治疗后，应用PET-CT进行中期和末期评估。疗效评估标准参照2014年Lugano国际工作组标准[13]。28例患者中，17例完成6个疗程BR方案后接受了利妥昔单抗单药维持，3例尚未进入利妥昔单抗维持治疗阶段，8例因各种原因未接受利妥昔单抗维持治疗。

3. 随访：通过查阅患者住院或门诊病历随访及电话随访。末次随访时间为2021年7月30日。OS时间定义为自本次疾病明确进展或复发（即BR方案治疗前FL进展或复发的时间）至因任何原因死亡或末次随访时间。PFS时间定义为自本次疾病明确进展或复发至再次进展/复发、死亡或末次随访时间。

4. 统计学处理：应用SPSS 26.0软件进行统计学分析。采用Kaplan-Meier法描绘生存曲线，计数资料用例数（百分比）表示，计量资料用中位数（范围）表示。采用*χ*^2^检验或Fisher精确检验法进行组间比较，双侧*P*<0.05为差异有统计学意义。

## 结果

1. 一般资料：28例患者中男11例，女17例，中位年龄54（31～76）岁。年龄大于60岁者10例（35.8％）。有B症状（发热、盗汗、体重减轻）者6例（21.4％）。Ann Arbor分期Ⅲ/Ⅳ期27例（96.4％），FILPI2高危（3～5分）7例（25％），美国东部肿瘤协作组（ECOG）体能状态评分≥2分者3例（10.7％）。累及≥2个结外器官的患者5例（17.9％），骨髓受累患者18例（64.3％）。血清乳酸脱氢酶（LDH）水平升高（>192 U/L）患者11例（39.3％），β_2_-MG升高（>3 mg/L）患者9例（32.1％），贫血（HGB<120 g/L）患者8例（28.6％）。淋巴结直径大于6 cm的患者4例（14.3％），复发距末次治疗时间<1年的患者17例（60.7％），病理分级1～2级患者15例（53.6），3a级患者13例（46.4％）。BCL-2阳性患者14例（50％），POD24患者20例（71.4％）。既往接受过R-CHOP方案治疗者9例，接受过R2方案（利妥昔单抗+来那度胺）治疗者19例。

2. 基因突变特征：应用靶向测序方法检测28例复发FL患者的肿瘤组织[14]，24例（85.7％）患者发现基因突变。表观遗传学基因突变发生率高：CREBBP突变20例（71.4％），KMT2D突变18例（64.3％），HIST1H1E突变5例（17.9％），EZH2突变4例（14.3％），ARID1A突变4例（14.3％），EP300突变3例（10.7％），KMT2C突变2例（7.1％）。其他突变频率大于5％的基因有：STAT6突变4例（14.3％），TNFRSF14突变4例（14.3％），CARD11突变4例（14.3％），SOCS1突变3例（10.7％），FOXO1突变2例（7.1％），CIITA突变2例（7.1％），B2M突变2例（7.1％），DTX1突变2例（7.1％）。

3. 疗效：28例患者中位接受BR方案6（3～6）个疗程，25例完成了6个疗程BR方案治疗。1例完成了3个疗程BR方案治疗，2例完成了5个疗程BR方案治疗，3例尚未完成6个疗程治疗的患者均已完成了中期评估。可评估的28例患者中，18例（64.3％）达完全代谢学缓解（CMR），10例达部分代谢学缓解（PMR），客观缓解率（ORR）为100％。对可能影响患者达到CMR的临床因素进行分析，达到与未达到CMR组的差异均无统计学意义（*P*值均>0.05）（表1）。对肿瘤基因突变累及的信号通路进行分析，达到与未达到CMR组的差异均无统计学意义（*P*值均>0.05）（表2）。

**表1:** 影响复发/难治滤泡性淋巴瘤患者达到完全代谢学缓解（CMR）的临床因素分析［例（％）］

因素	达到CMR（18例）	未达到CMR（10例）	*P*值
病理分级			1.000
1～2级	10（66.7）	5（33.3）	
3a级	8（61.5）	5（38.5）	
进展/复发时间			1.000
≤1年	11（64.8）	6（35.2）	
>1年	7（63.6）	4（36.4）	
既往治疗方案			1.000
R-CHOP方案	6（66.7）	3（33.3）	
R2方案	12（63.2）	7（36.8）	
大包块			0.601
有	2（50.0）	2（50.0）	
无	16（66.7）	8（33.3）	
结外器官受累			1.000
≥2个	3（60.0）	2（40.0）	
<2个	15（65.2）	8（34.8）	
骨髓累及			0.097
有	14（77.8）	4（22.2）	
无	4（40.0）	6（60.0）	
POD24			1.000
是	13（65.0）	7（35.0）	
否	5（62.5）	3（37.5）	
FLIPI2			1.000
0～2分	13（61.9）	8（38.1）	
3～5分	5（71.4）	2（28.6）	
BCL-2			0.695
阳性	10（71.4）	4（28.6）	
阴性	8（57.1）	6（42.9）	

注：R-CHOP：利妥昔单抗+环磷酰胺+多柔比星+长春新碱+泼尼松；R2：利妥昔单抗+来那度胺；POD24：治疗后2年内进展；FLIPI2：滤泡性淋巴瘤国际预后指数2

**表2:** 影响复发/难治滤泡性淋巴瘤患者达到完全代谢学缓解（CMR）的基因突变信号通路分析［例（％）］

基因突变信号通路	达到CMR（18例）	未达到CMR（10例）	*P*值
组蛋白乙酰化			0.207
有突变	15（71.4）	6（29.6）	
无突变	3（42.9）	4（57.1）	
组蛋白/DNA甲基化			0.694
有突变	13（68.4）	6（31.6）	
无突变	5（55.6）	4（44.4）	
细胞周期/p53通路			1.000
有突变	6（66.7）	3（33.3）	
无突变	12（63.2）	7（36.8）	
干扰素-γ通路			0.674
有突变	4（57.1）	3（42.9）	
无突变	14（66.7）	7（33.3）	
JAK-STAT			1.000
有突变	4（66.7）	2（33.3）	
无突变	14（63.7）	8（36.3）	
T细胞活化通路			1.000
有突变	3（60.0）	2（40.0）	
无突变	15（65.2）	8（34.8）	
BCR/NF-κB通路			1.000
有突变	3（60.0）	2（40.0）	
无突变	15（65.2）	8（34.8）	
Wnt 信号通路			1.000
有突变	3（60.0）	2（40.0）	
无突变	15（65.2）	8（34.8）	
染色质重组			1.000
有突变	3（75.0）	1（25.0）	
无突变	15（62.5）	9（37.5）	

4. 生存情况：截至2021年7月30日，中位随访时间为12（4～24）个月，无患者出现疾病进展或死亡。1年OS率为100％，1年PFS率为100％。

5. 不良反应：28例患者中血液学不良事件包括中性粒细胞减少（发生率46.4％，Ⅲ级发生率14.3％，Ⅳ级发生率7.1％）、血小板下降（发生率17.9％，均为Ⅰ~Ⅱ级），贫血（发生率14.3％，均为Ⅰ~Ⅱ级），7例（25％，7/28）出现Ⅲ/Ⅳ级非血液学不良事件，经过对症支持治疗后均改善。非血液学不良事件包括恶心（46.4％）、呕吐（17.9％）、发热（3.6％）、肺部感染（3.6％）、皮疹（21.4％）。

10例患者年龄>60岁，化疗耐受性好。其中5例（50％）患者出现血液学不良反应，3例（30％）出现Ⅲ/Ⅳ级中性粒细胞下降，1例（10％）发生肺部感染，对症治疗后均改善。非血液学不良事件以恶心为主。血液学不良反应和非血液学不良反应与年轻患者无明显差异。

## 讨论

FL是较常见的小B细胞来源的NHL，指南推荐，对于FL病理1～3a级的Ⅰ～Ⅱ期患者建议采用放疗或放疗±化疗[15]。Ⅲ/Ⅳ期患者应用免疫化疗或者靶向治疗可以控制疾病，但不能治愈，复发不可避免[16]。

本研究纳入28例经R-CHOP/R2方案治疗后复发或进展的病理分级1～3a级患者，应用BR方案治疗后，18例（64.3％）达CMR，10例达PMR，ORR为 100％，1年OS率和1年PFS率均为100％，疗效显著，与国外的报道类似。BRIGHT多中心Ⅲ期临床试验纳入447例初治惰性NHL和套细胞性淋巴瘤（MCL）患者，进行1∶1随机分组，与R-CHOP/R-CVP方案组相比，BR方案组患者有更高ORR（97％对91％，*P*＝0.010）和更高的CR率（31％对25％，*P*＝0.022）[17]。BRIGHT研究是2009−2012年进行的，患者治疗后的缓解情况主要通过增强CT进行评估。GALLIUM研究结果提示对于免疫化疗后的FL，PET/CT是一种比增强CT更好的疗效评估方法[18]。日本进行的一项评价BR方案治疗复发/难治性惰性B-NHL的研究中，复发/难治性FL患者有很高的有效率，ORR为95％，CR率为80％，与我们的研究结果相似[19]。

R-CHOP/R-CVP方案治疗时，FL的病理分级（3a级）、骨髓受累、大包块、结外器官受累（≥2个）、POD24、FLIPI2高危、BCL-2阳性等临床因素均影响治疗效果，骨髓受累、大包块等是独立的预后不良因素[20]–[21]。本组28例复发进展的FL患者中，既往接受R-CHOP方案化疗的患者9例，R2方案化疗的患者19例，BR方案仍获得较高的CMR率，R-CHOP方案与R2方案治疗患者的CMR差异无统计学意义。该结果提示，对于R-CHOP/R2方案治疗后进展的FL患者，即使对利妥昔单抗耐药，联合苯达莫司汀后仍可获得较高的CMR率。对可能影响疗效的临床因素进行分析，病理分级（3a级）、骨髓受累、大包块、结外器官受累（≥2个）、POD24、FLIPI2高危、BCL2阳性均不影响BR方案的治疗效果。

有研究提出CREBBP和EP300乙酰转移酶的失活突变是FL中最常见的遗传改变[22]。也有报道提出CREBBP和KMT2D突变在FL中占优势[23]。Sorigue等[24]报道，在25例复发FL患者中，最常见的突变基因是CREBBP、EP300、ARID1A和IRF8，EP300的突变率较既往报道高10％～15％，且ARID1A、MEF2B、EP300、FOXO1、CREBBP和CARD11突变的FL患者应用免疫化疗治疗预后差[25]。

本研究中28例复发进展FL应用靶向测序方法，24例（85.7％）发现基因突变。其中表观遗传学基因突变最为常见，包括CREBBP、KMT2D、HIST1H1E、EZH2、EP300。m7-FLIPI模型中的基因ARID1A、CARD11、FOXO1均检测到突变，未检测到MEF2B突变。应用BR方案治疗后，无论是乙酰化基因突变者还是甲基化基因发生突变者，均能获得较高的CMR率，即使存在其他通路基因突变的FL患者，也能通过BR方案治疗获得60％以上的CMR率。

不良反应方面，BRIGHT研究中BR方案组（221例）不良事件发生率分别为：白细胞减少38％、中性粒细胞减少44％、HGB下降3％、PLT下降7％、感染9％、肺炎3％、药物过敏2％、呕吐3％、高血糖<1％。BRIGHT研究报道了477例一线治疗应用BR方案的初治惰性NHL和MCL患者，第二肿瘤是应用BR方案治疗的长期生存患者需要关注的事件，本研究观察时间较短，因此未观察到第二肿瘤的发生[17]。日本的一项研究纳入应用BR方案治疗的53例复发难治性惰性B-NHL和MCL患者，BR方案治疗耐受性良好，主要不良反应为骨髓抑制。3～4级白细胞减少和中性粒细胞减少的发生率分别为42％和40％，无Ⅴ级毒性[19]。Hess等[26]报道的Ⅰ/Ⅱ期临床试验应用西罗莫司联合BR方案治疗复发难治性FL和MCL，共纳入39例（29例MCL，10例FL）患者。Ⅲ～Ⅳ级非血液学不良事件罕见，高血糖3例，血管性水肿2例。9例患者出现Ⅲ～Ⅳ级感染性并发症。血液学Ⅲ～Ⅳ级不良反应包括白细胞减少22例，中性粒细胞减少18例，淋巴细胞减少16例，血小板减少14例。本研究结果显示，复发难治FL患者应用BR方案产生的不良反应可耐受，28例患者的血液学不良事件包括中性粒细胞减少、血小板下降和贫血。25％的患者出现Ⅲ/Ⅳ级非血液学不良事件，经过对症支持治疗后均改善。不良反应发生率与国外报道相似。本组患者有10例年龄>60岁，BR方案耐受性好。其中5例患者出现血液学不良反应，3例出现Ⅲ/Ⅳ级中性粒细胞减少，1例发生肺部感染。

综上所述，复发FL患者应用BR方案的疗效较好，不良反应可耐受。CREBBP、EP300等预后不良的基因突变不影响BR方案的疗效。本研究是单中心、小样本量的病例报道，还需要大规模前瞻性临床研究数据验证。

## References

[1] Hiddemann W, Kneba M, Dreyling M (2005). Frontline therapy with rituximab added to the combination of cyclophosphamide, doxorubicin, vincristine, and prednisone (CHOP) significantly improves the outcome for patients with advanced-stage follicular lymphoma compared with therapy with CHOP alone: results of a prospective randomized study of the German Low-Grade Lymphoma Study Group[J]. Blood.

[2] Walewski J, Paszkiewicz-Kozik E, Michalski W (2020). First-line R-CVP versus R-CHOP induction immunochemotherapy for indolent lymphoma with rituximab maintenance. A multicentre, phase III randomized study by the Polish Lymphoma Research Group PLRG4[J]. Br J Haematol.

[3] Johnson PW, Rohatiner AZ, Whelan JS (1995). Patterns of survival in patients with recurrent follicular lymphoma: a 20-year study from a single center[J]. J Clin Oncol.

[4] 王 楠, 许 彭鹏, 王 黎 (2019). 利妥昔单抗联合化疗治疗229例滤泡性淋巴瘤患者的预后研究[J]. 中华血液学杂志.

[5] 许 彭鹏, 钱 樱, 陈 秋生 (2017). 滤泡性淋巴瘤国际预后指数2在利妥昔单克隆抗体维持治疗滤泡性淋巴瘤患者中的预后意义[J]. 中国实验血液学杂志.

[6] Casulo C, Barr PM (2019). How I treat early-relapsing follicular lymphoma[J]. Blood.

[7] Pastore A, Jurinovic V, Kridel R (2015). Integration of gene mutations in risk prognostication for patients receiving first-line immunochemotherapy for follicular lymphoma: a retrospective analysis of a prospective clinical trial and validation in a population-based registry[J]. Lancet Oncol.

[8] MacDonald D, Prica A, Assouline S (2016). Emerging therapies for the treatment of relapsed or refractory follicular lymphoma[J]. Curr Oncol.

[9] Sakai R, Ohmachi K, Sano F (2019). Bendamustine-120 plus rituximab therapy for relapsed or refractory follicular lymphoma: a multicenter phase II study[J]. Ann Hematol.

[10] Flinn IW, van der Jagt R, Kahl B (2019). First-Line Treatment of Patients With Indolent Non-Hodgkin Lymphoma or Mantle-Cell Lymphoma With Bendamustine Plus Rituximab Versus R-CHOP or R-CVP: Results of the BRIGHT 5-Year Follow-Up Study[J]. J Clin Oncol.

[11] Swerdlow SH, Campo E, Pileri SA (2016). The 2016 revision of the World Health Organization classification of lymphoid neoplasms[J]. Blood.

[12] Morin RD, Mendez-Lago M, Mungall AJ (2011). Frequent mutation of histone-modifying genes in non-Hodgkin lymphoma[J]. Nature.

[13] Cheson BD, Fisher RI, Barrington SF (2014). Recommendations for initial evaluation, staging, and response assessment of Hodgkin and non-Hodgkin lymphoma: the Lugano classification[J]. J Clin Oncol.

[14] Green MR (2018). Chromatin modifying gene mutations in follicular lymphoma[J]. Blood.

[15] Dreyling M, Ghielmini M, Rule S (2021). Newly diagnosed and relapsed follicular lymphoma: ESMO Clinical Practice Guidelines for diagnosis, treatment and follow-up[J]. Ann Oncol.

[16] Batlevi CL, Sha F, Alperovich A (2020). Follicular lymphoma in the modern era: survival, treatment outcomes, and identification of high-risk subgroups[J]. Blood Cancer J.

[17] Flinn IW, van der Jagt R, Kahl B (2019). First-Line Treatment of Patients With Indolent Non-Hodgkin Lymphoma or Mantle-Cell Lymphoma With Bendamustine Plus Rituximab Versus R-CHOP or R-CVP: Results of the BRIGHT 5-Year Follow-Up Study[J]. J Clin Oncol.

[18] Trotman J, Barrington SF, Belada D (2018). Prognostic value of end-of-induction PET response after first-line immunochemotherapy for follicular lymphoma (GALLIUM): secondary analysis of a randomised, phase 3 trial[J]. Lancet Oncol.

[19] Matsumoto K, Takayama N, Aisa Y (2015). A phase II study of bendamustine plus rituximab in Japanese patients with relapsed or refractory indolent B-cell non-Hodgkin lymphoma and mantle cell lymphoma previously treated with rituximab: BRB study[J]. Int J Hematol.

[20] Matasar MJ, Luminari S, Barr PM (2019). Follicular Lymphoma: Recent and Emerging Therapies, Treatment Strategies, and Remaining Unmet Needs[J]. Oncologist.

[21] 杜 晓艳, 胡 凯, 赵 伟 (2018). 94例滤泡性淋巴瘤患者的临床特征及预后因素分析[J]. 中国实验血液学杂志.

[22] Meyer SN, Scuoppo C, Vlasevska S (2019). Unique and Shared Epigenetic Programs of the CREBBP and EP300 Acetyltransferases in Germinal Center B Cells Reveal Targetable Dependencies in Lymphoma[J]. Immunity.

[23] Korfi K, Ali S, Heward JA (2017). Follicular lymphoma, a B cell malignancy addicted to epigenetic mutations[J]. Epigenetics.

[24] Sorigue M, Oliveira A, Mercadal S (2019). m7FLIPI and targeted sequencing in high-risk follicular lymphoma[J]. Hematol Oncol.

[25] Lockmer S, Ren W, Brodtkorb M (2020). M7-FLIPI is not prognostic in follicular lymphoma patients with first-line rituximab chemo-free therapy[J]. Br J Haematol.

[26] Hess G, Wagner K, Keller U (2020). Final Results of a Phase I/II Trial of the Combination Bendamustine and Rituximab With Temsirolimus (BeRT) in Relapsed Mantle Cell Lymphoma and Follicular Lymphoma[J]. Hemasphere.

